# Appendicitis in De Garengeot Hernia: A Rare Presentation

**DOI:** 10.7759/cureus.68352

**Published:** 2024-08-31

**Authors:** Ahmed A Alkhalifa, Mohammed Alabbad, Hassan M Alshahri, Buthainah Aljughaiman, Hazem F Helal, Abdulmajeed M Al Omair, Othman Almohammedsaleh, Nizar M Amro, Ahmed M Odeh

**Affiliations:** 1 General and Laparoscopic Surgery, Al Ahsa Health Cluster, Al Ahsa, SAU; 2 General Surgery, Al Ahsa Health Cluster, Al Ahsa, SAU; 3 Radiology, King Fahad Hospital - Hofuf, Hofuf, SAU; 4 General Surgery, New You Medical Center, Riyadh, SAU

**Keywords:** de garengeot hernia, de garengeot hernia with appendicitis, amyand’s hernia, appendicitis in femoral hernia, femoral hernia

## Abstract

De Garengeot hernia, a rare clinical entity characterized by the presence of the appendix within a femoral hernia sac, is a challenging condition often requiring prompt surgical intervention. Fewer than 100 cases have been reported in the literature containing appendicitis in de Garengeot hernia. We present a case of a 40-year-old female with a preoperatively diagnosed De Garengeot hernia with appendicitis. The patient presented with a painful and irreducible right groin swelling, and imaging revealed a femoral hernia containing an inflamed appendix. Urgent surgical intervention was undertaken, involving right femoral hernia repair and appendectomy. The surgical procedure, utilizing the infra-inguinal approach, proceeded uneventfully, with the patient recovering well postoperatively. De Garengeot hernia is a rare clinical presentation with no established treatment guidelines. Surgical management typically involves hernia repair and appendectomy, with the choice of repair method dependent on intraoperative findings. The use of prosthetic mesh in contaminated fields remains controversial, with considerations for infection risk and patient outcomes. Early diagnosis and appropriate surgical intervention are essential in managing De Garengeot hernia to prevent complications and ensure favourable patient outcomes. This case underscores the importance of recognizing and effectively treating this uncommon surgical condition.

## Introduction

A femoral hernia is characterized by the protrusion of abdominal contents through the femoral ring, which is located medial to the femoral vein and beneath the external inguinal ring. Although femoral hernias account for only 3% of all hernias, they are more frequently observed in females and present a significant risk of strangulation due to the constricted nature of the femoral neck [1].

In 0.5%-5% of cases, the appendix may herniate through the femoral canal, a condition known as De Garengeot hernia. The occurrence of appendicitis within a De Garengeot hernia is exceedingly rare, with reported incidence rates ranging from 0.08% to 0.13%. De Garengeot hernia represents an uncommon clinical entity with no established guidelines regarding the optimal management approach. It is crucial to distinguish this condition from an Amyand's hernia, which similarly involves the appendix but within an inguinal hernia [2]. In this report, we present a case of a De Garengeot hernia that was diagnosed preoperatively and discuss implications for enhancing the understanding of this rare condition.

## Case presentation

A 40-year-old female presented to the Emergency Room with a right groin swelling persisting for three days. She reported a similar incident a year ago that resolved spontaneously. Over the past two days, the swelling became painful and irreducible. The patient denied experiencing abdominal pain, nausea, vomiting, loss of appetite, fever, changes in bowel habits, or a history of trauma. She mentioned handling heavy workloads while caring for her handicapped child.

Her medical and surgical history was unremarkable.

**Figure 1:**
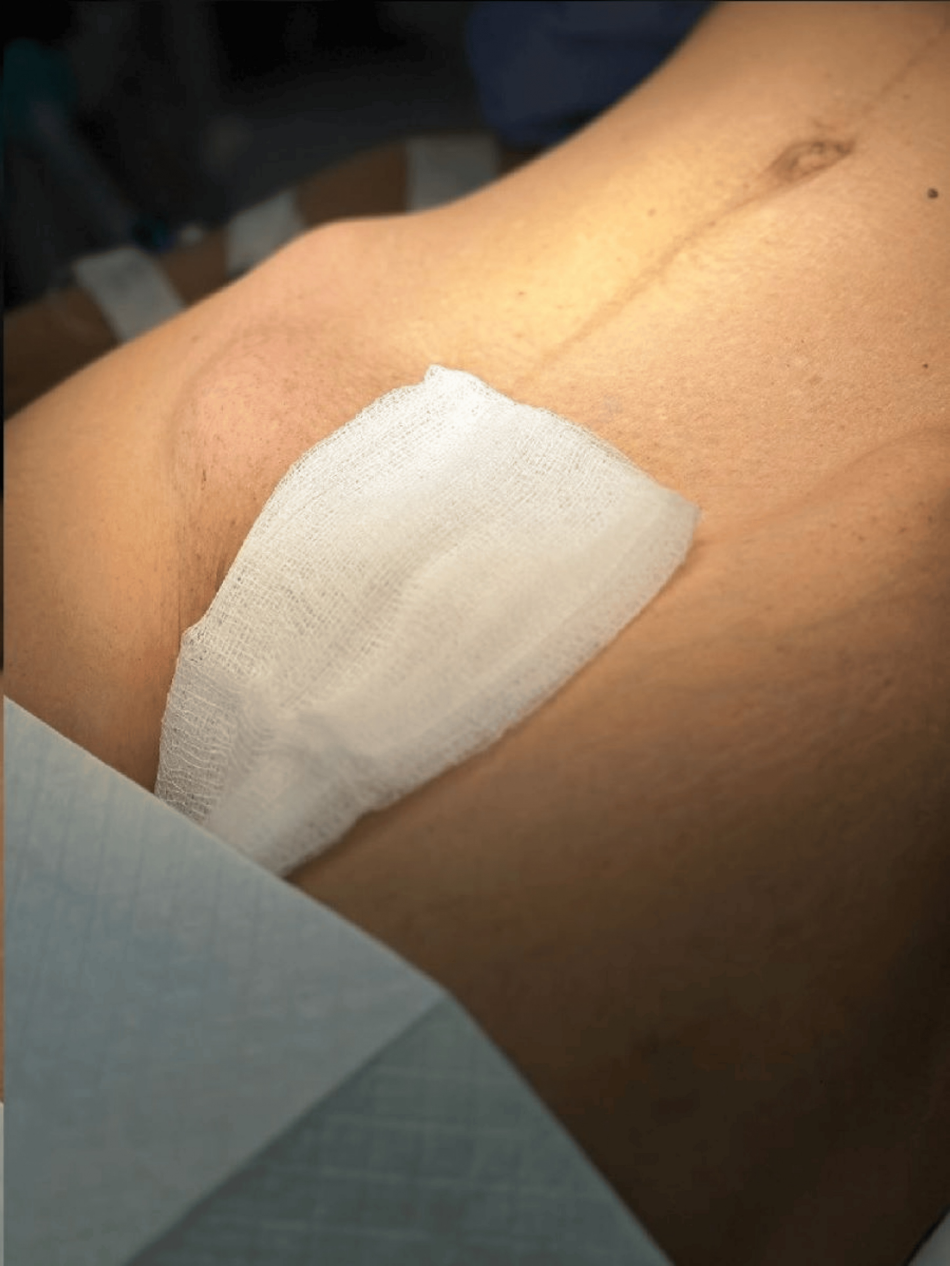
Preoperative picture showing right groin swelling at presentation.

On physical examination, the patient appeared underweight, afebrile, and hemodynamically stable. Abdominal assessment revealed a soft, lax, non-distended abdomen with a palpable lump below the right inguinal ligament. The swelling was 4x2 cm firm, irreducible, tender, lacking a cough impulse, and devoid of associated skin changes (Figure 1).

**Figure 2:**
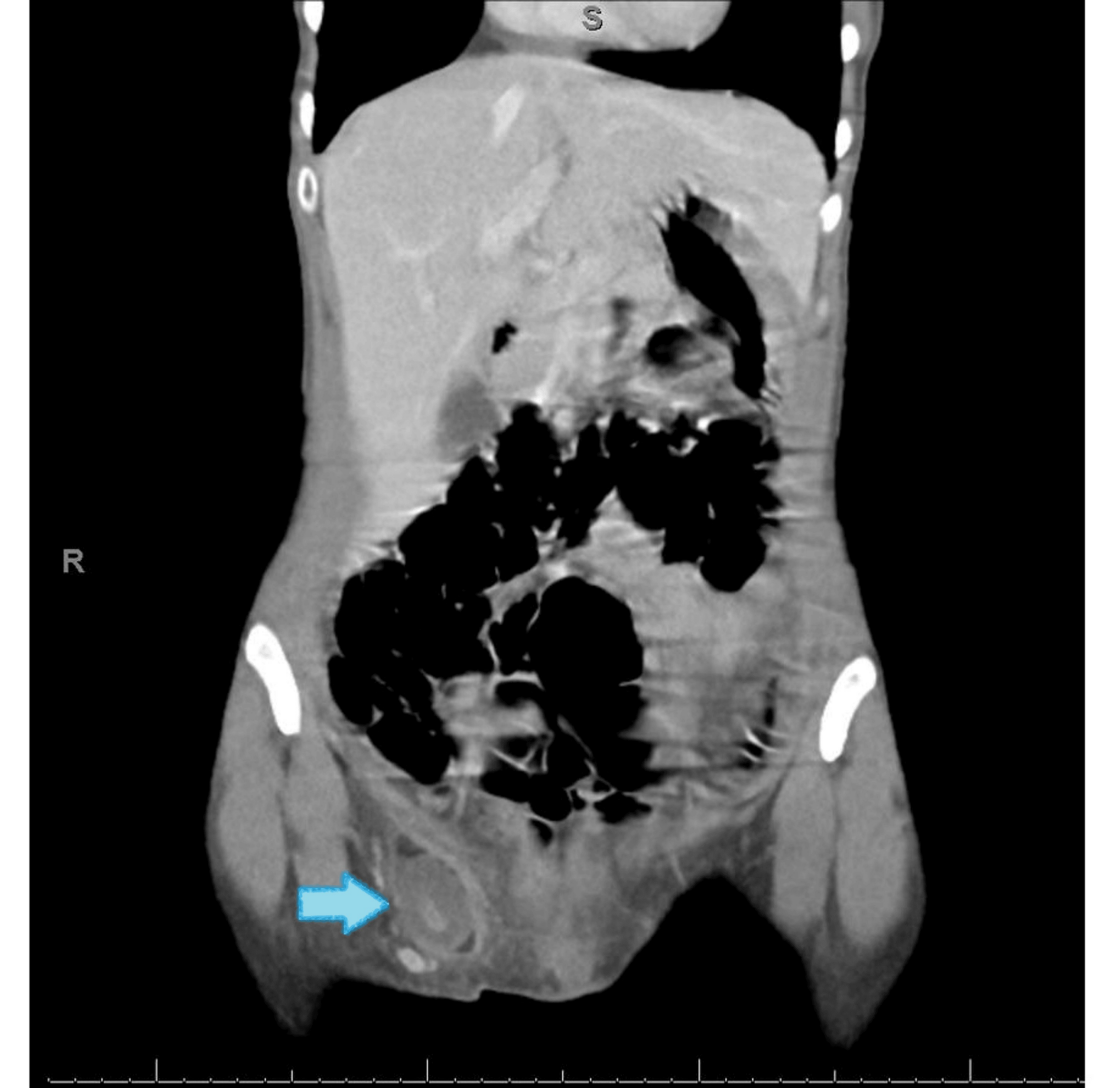
Abdominal CT scan with contrast revealing a femoral hernia on the right side containing the inflamed, dilated, 7 mm appendix, swirling fluid, and surrounding stranding indicative of inflammatory changes.

Laboratory investigations returned within normal parameters. An abdominal CT scan with contrast was performed, revealing a femoral hernia on the right side containing an inflamed appendix as it was dilated 7 mm, enhancing wall, swirling fluid, and surrounding stranding indicative of inflammatory changes (Figure 2).

The decision was made to proceed with the right femoral hernia repair and appendectomy in the operating theatre. The patient was prepared by maintaining nothing by mouth (NPO) status, ensuring adequate hydration, and administering prophylactic antibiotics and analgesia.

**Figure 3:**
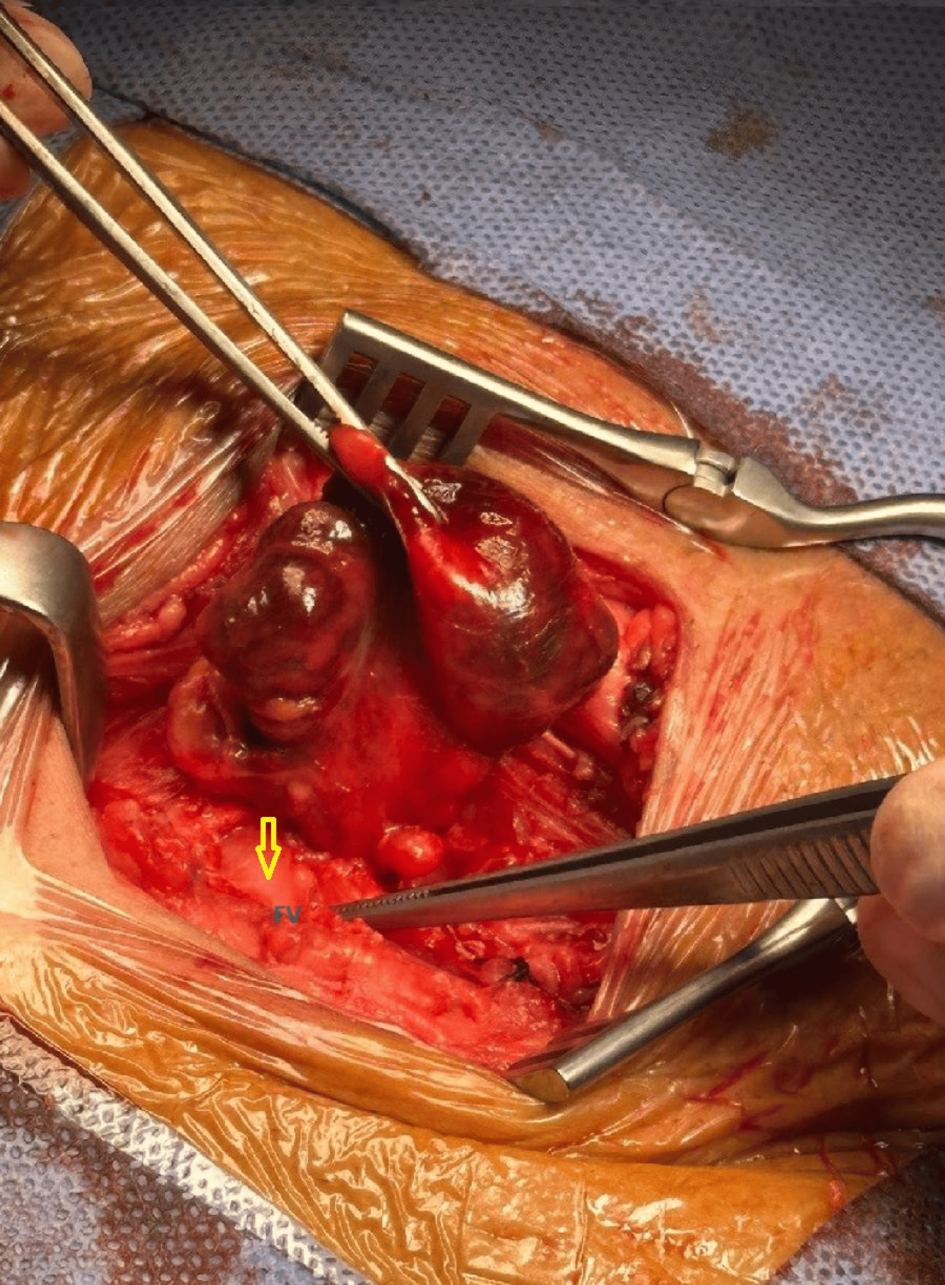
Intraoperative picture showing hernial sac and secured femoral vein (FV).

Using the infra-inguinal approach (Lockwood approach), the femoral hernia sac was dissected from dense adhesions (Figure 3).

**Figure 4:**
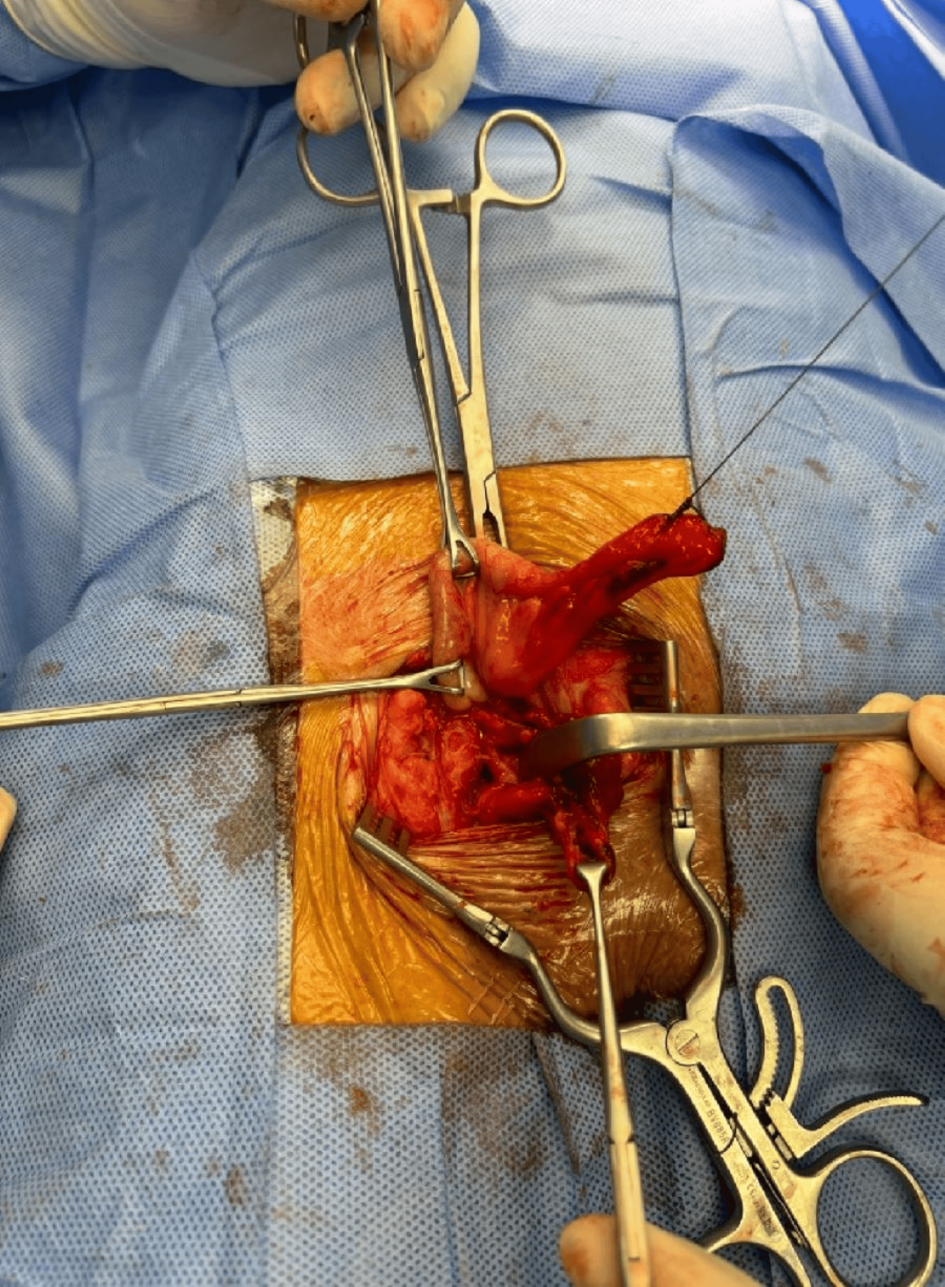
Intraoperative picture showing hernial sac content/inflamed appendix.

The femoral vein was identified and preserved with ligation of collateral veins to free the sac. Due to a narrow femoral neck, a small incision of the inguinal ligament was necessary to access the content of the irreducible sac. The sac was opened, and the appendix was inflamed with a dusky bluish tip and adherent within the sac (Figure 4).

**Figure 5:**
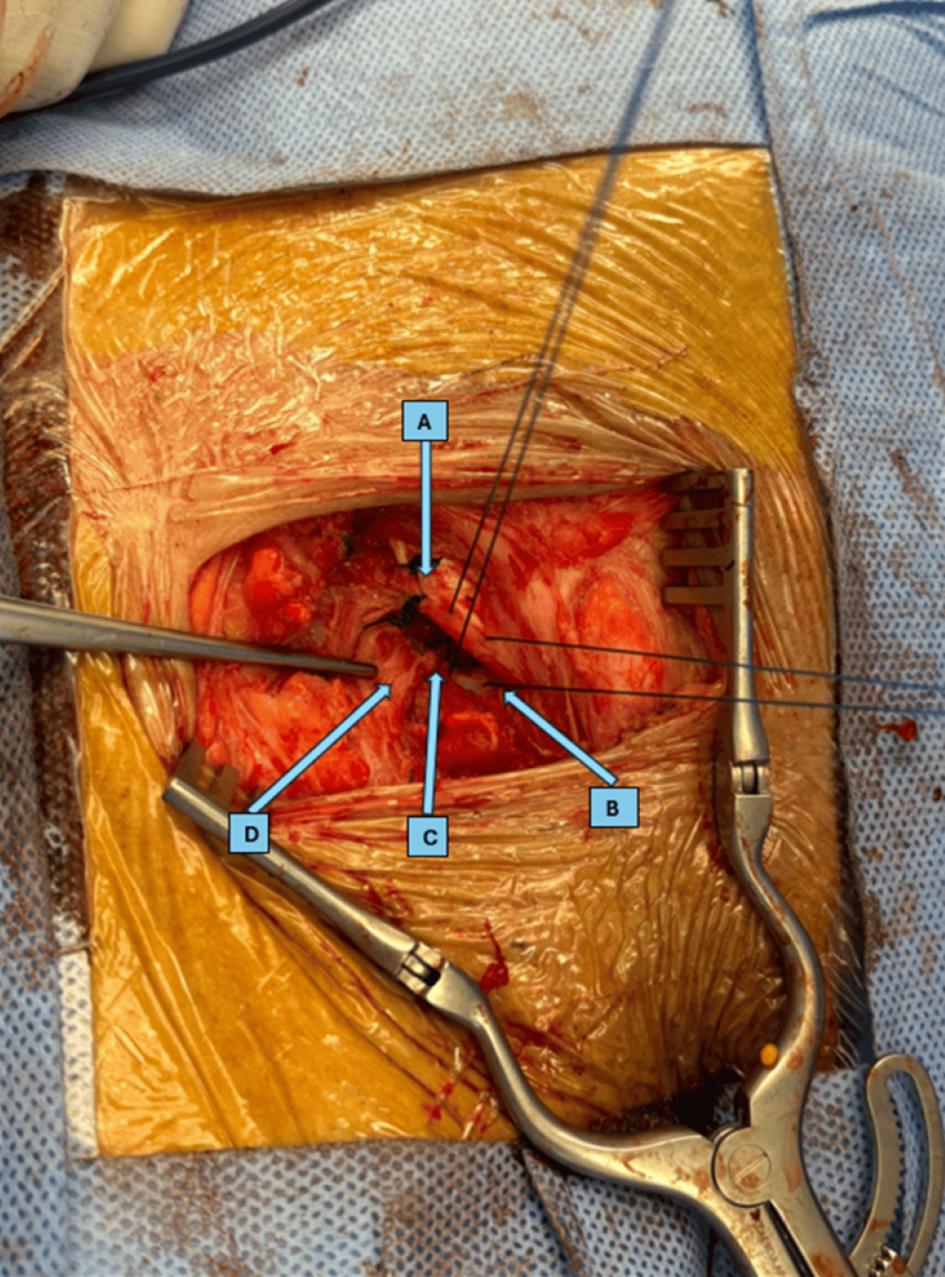
Preclosure anatomy. A) Inguinal ligament, B) lacunar ligament, C) pectineal ligament, and D) femoral vein

**Figure 6:**
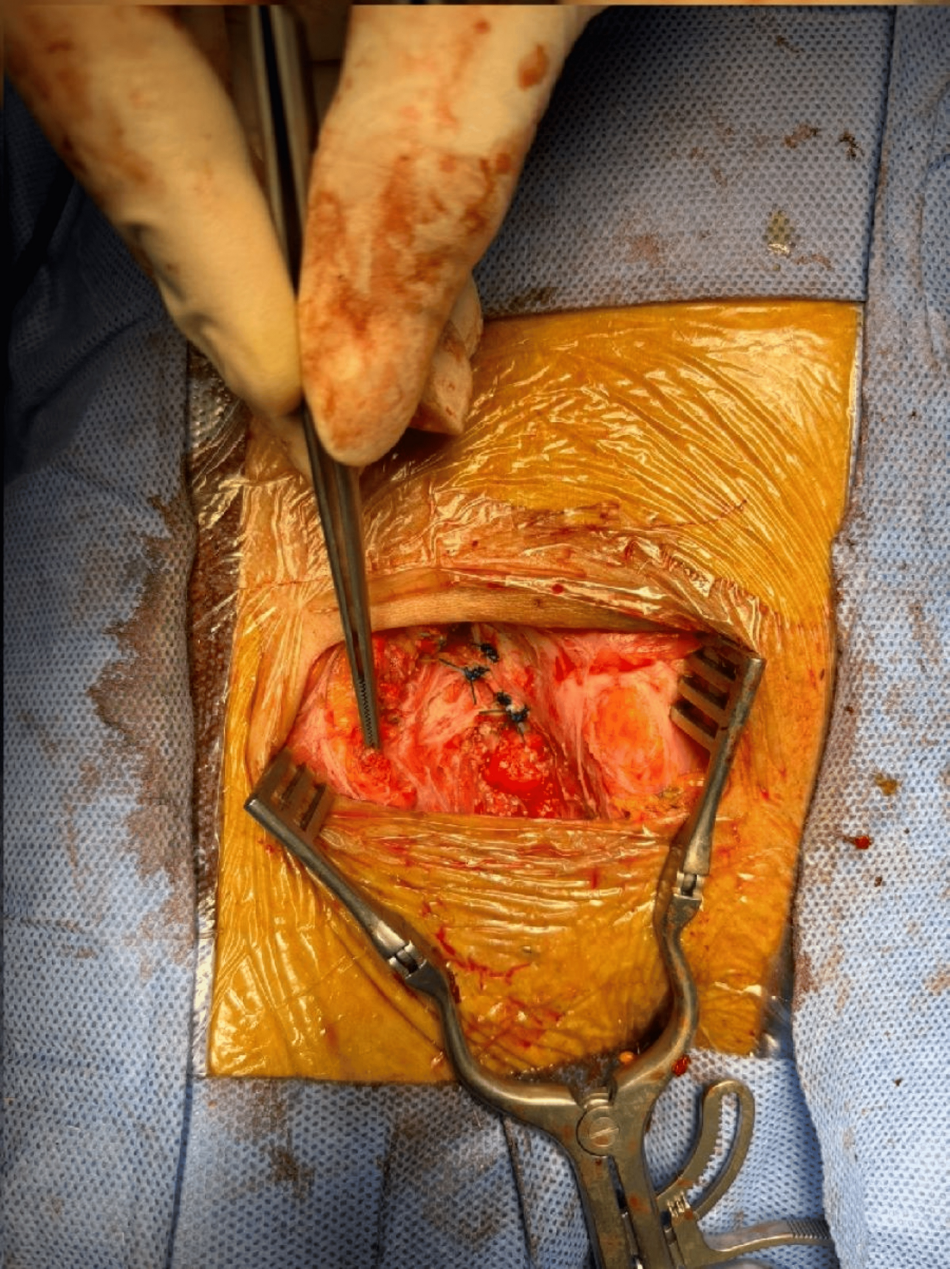
Intraoperative picture showing the primary closure of the femoral canal.

An appendectomy was performed, followed by closure and reduction of the femoral hernia sac. Appling McVay, the inguinal ligament was repaired using prolene 0, and the femoral defect was closed by approximating the Pectineal ligament to the Inguinal ligament prolene 0 ensuring no narrowing of the femoral vein (Figures 5-6).

**Figure 7:**
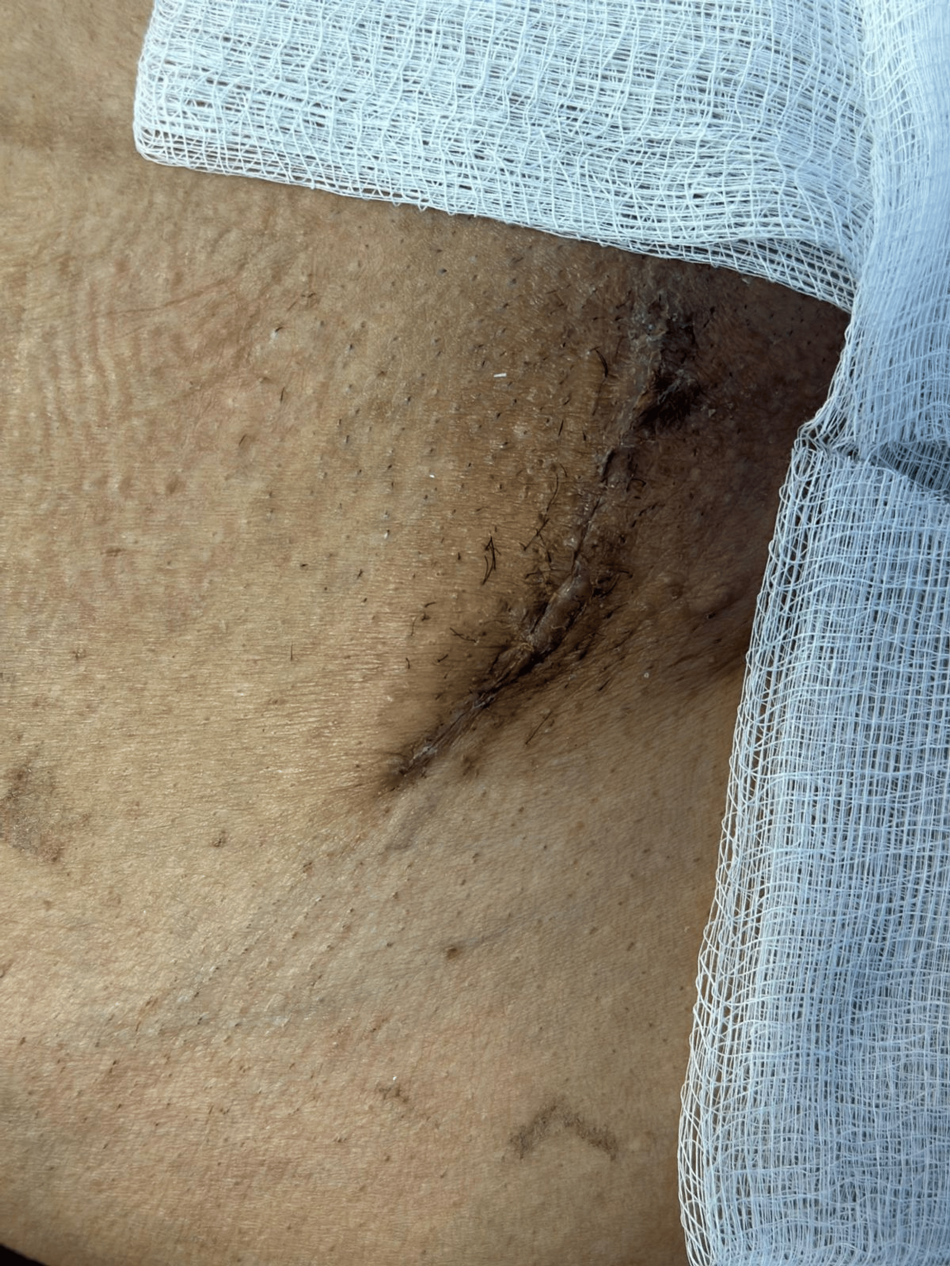
Follow-up after operation in two weeks at the clinic and the wound healed without complication or recurrence.

The surgical procedure proceeded without complications, and the patient recovered well postoperatively, initiating diet intake, and passing bowel movements. The wound exhibited no signs of infection, and the patient was discharged in good condition on the second day following the operation. The patient was seen after two weeks at the clinic without complications (Figure 7).

## Discussion

Hernias represent a prevalent surgical condition, with approximately 75% occurring in the inguinal region and 3% in the femoral region [3,4]. A particularly rare form of hernia, known as De Garengeot hernia, is characterized by a femoral hernia that contains an appendix within the sac [1]. In the literature, less than 100 cases of this condition were reported. The incidence of an appendix within a femoral hernia is reported to be between 0.8% and 1% of all femoral hernias; furthermore, the presence of acute appendicitis in the femoral hernia is even rarer, estimated at around 0.08%-0.13% [1,4,5]. This condition is more prevalent in women than in men, with a reported ratio of 3:1, which corresponds to the higher prevalence of femoral hernias in females [3,4]. The condition was first described by Rene Jacques Croissant de Garengeot in 1731, and the first recorded appendectomy in such a case was performed by Hevin in 1785 [3,5].

Clinically, De Garengeot hernia typically presents as an incarcerated hernia, which is characterized by painful, irreducible groin swelling and may be associated with signs of inflammation [1,3,6,7]. Patients may also present with fever and symptoms indicative of obstructive acute abdomen [6,7]. Laboratory findings in these cases are generally nonspecific [7]. The differential diagnosis should include considerations such as incarcerated inguinal hernia, Richter or Littre hernia, lymph node, aneurysm, adnexa, lipomas, lymphomas, abscesses, and tuberculosis [8]. Preoperative diagnosis of De Garengeot hernia is often challenging, and acute appendicitis within the sac is usually confirmed intraoperatively [3,8]. While few preoperative diagnoses have been made using CT imaging, the typical findings on a CT scan may include a hernia sac containing a tubular structure, fat stranding, and inferiorly positioned cecum; ultrasound can also be useful in identifying bowel contents within the hernia sac [8]. Abdominal X-rays are generally can be used to identify small bowel obstruction. Despite imaging findings, the surgeon's clinical judgment should ultimately guide the final diagnosis and treatment decision [6]. In the case presented, a CT scan revealed a right-sided femoral hernia containing a 7 mm appendix, with swirling fluid and surrounding fat stranding suggestive of inflammatory changes. Discussion with the radiologist confirmed the diagnosis of femoral hernia with acute appendicitis.

The management of De Garengeot hernia complicated by appendicitis is a surgical emergency [3,9,10]. Given the rarity of this condition, there are no standardized treatment guidelines until now [4,5,8]. Most of the literature advocates for the reduction of the appendix when it is neither necrotic nor inflamed, followed by hernia repair [9]. Herniorrhaphy is indicated when an appendectomy is necessary, and it is acceptable to perform an appendectomy through the hernia sac, though the use of a laparoscopic approach in such cases remains controversial [6,7,10]. The use of prosthetic mesh for incarcerated inguinal hernias requiring bowel resection may be adaptable for De Garengeot hernia [11,12]. Current consensus suggests that mesh can be safely applied in the absence of abscess or perforation, without increasing the risk of infection or recurrence [6,13]. In the case presented, intraoperative findings included an inflamed appendix and a dusky hernia sac, making herniorrhaphy the most appropriate choice for repair, and no mesh was applied, consistent with established treatment strategies for such cases.

Wound complications can arise, with a higher prevalence observed among older patients due to delays in diagnosis and management. The infection rate can escalate to as much as 29%. Although rare, severe complications such as necrotizing fasciitis and mortality have been documented [3,7,10,11].

## Conclusions

De Garengeot hernia is a rare and challenging condition that requires prompt surgical intervention. The diagnosis is often made intraoperatively, and treatment typically involves hernia repair and appendectomy. The use of prosthetic materials in repair should be carefully considered based on the presence of contamination and the patient's overall condition. Early diagnosis and appropriate surgical management are crucial to prevent complications and ensure favourable outcomes.
